# Examining the role of COVID-19 testing availability on intention to isolate: A Randomized hypothetical scenario

**DOI:** 10.1371/journal.pone.0262659

**Published:** 2022-02-02

**Authors:** Justin C. Zhang, Katherine L. Christensen, Richard K. Leuchter, Sitaram Vangala, Maria Han, Daniel M. Croymans

**Affiliations:** 1 David Geffen School of Medicine at UCLA, Los Angeles, California, United States of America; 2 Kelley School of Business, Indiana University, Bloomington, Indiana, United States of America; 3 Department of Medicine, David Geffen School of Medicine, University of California, Los Angeles, Los Angeles, California, United States of America; 4 Department of Medicine Statistics Core, David Geffen School of Medicine, University of California, Los Angeles, Los Angeles, California, United States of America; IAVI, UNITED STATES

## Abstract

**Background:**

Little information exists on how COVID-19 testing influences intentions to engage in risky behavior. Understanding the behavioral effects of diagnostic testing may highlight the role of adequate testing on controlling viral transmission. In order to evaluate these effects, simulated scenarios were conducted evaluating participant intentions to self-isolate based on COVID-19 diagnostic testing availability and results.

**Methods:**

Participants from the United States were recruited through an online survey platform (Amazon Mechanical Turk) and randomized to one of three hypothetical scenarios. Each scenario asked participants to imagine having symptoms consistent with COVID-19 along with a clinical diagnosis from their physician. However, scenarios differed in either testing availability (testing available v. unavailable) or testing result (positive v. negative test). The primary outcome was intention to engage in high-risk COVID-19 behaviors, measured using an 11-item mean score (range 1–7) that was pre-registered prior to data collection. Multi-variable linear regression was used to compare the mean composite scores between conditions. The randomized survey was conducted between July 23^rd^ to July 29^th^, 2020.

**Results:**

A total of 1400 participants were recruited through a national, online, opt-in survey. Out of 1194 respondents (41.6% male, 58.4% female) with a median age of 38.5 years, participants who had no testing available in their clinical scenario showed significantly greater intentions to engage in behavior facilitating COVID-19 transmission compared to those who received a positive confirmatory test result scenario (mean absolute difference (SE): 0.14 (0.06), *P* = 0.016), equating to an 11.1% increase in mean score risky behavior intentions. Intention to engage in behaviors that can spread COVID-19 were also positively associated with male gender, poor health status, and Republican party affiliation.

**Conclusion:**

Testing availability appears to play an independent role in influencing behaviors facilitating COVID-19 transmission. Such findings shed light on the possible negative externalities of testing unavailability.

**Trial registration:**

*Effect of Availability of COVID-19 Testing on Choice to Isolate and Socially Distance*, NCT04459520, https://clinicaltrials.gov/ct2/show/NCT04459520.

## Introduction/Background

The United States has reported more cases of COVID-19 than any other country in the world [1]. One of the factors contributing to the spread of the SARS-Cov-2 virus has been inadequate self-isolation and engagement in behaviors associated with high risks of viral transmission [2]. Control of the virus has been further complicated by a polarized political landscape and longstanding sociodemographic disparities which not only limit access to healthcare, but also one’s resources and ability to self-isolate [3, 4].

While previous studies conducted during the COVID-19 pandemic as well as other infectious outbreaks have identified a number of factors associated with self-isolation behavior (e.g., demographics, vulnerability, political beliefs, fear), few studies have explored how COVID-19 testing availability directly influences intentions to engage in behaviors which facilitate viral transmission [5–7]. Insight into this relationship would inform the public health response to both the current and future outbreaks. To this end, we conducted a web-based survey with randomized, hypothetical vignettes evaluating the impact of different testing scenarios on risky behavioral intentions in those presumed to have COVID-19.

## Methods

### Study participants

Study participants were recruited from Amazon Mechanical Turk (MTurk), an online survey platform found to yield results comparable to that of traditional sampling means [8]. Participants were included in the final analysis if they were U.S. residents (defined by reporting a 5-digit zip code which corresponded to a U.S. geographic region), over 18 years of age (based on their survey response), and passed all attention checks. Surveys were completed in Qualtrics. This study received IRB exemption from the UCLA Institutional Review Board.

### Design

Eligible participants were invited to complete a 5-question, pre-test Qualtrics survey through Amazon Mechanical Turk, assessing Theory of Planned Behavior (TPB) construct response (**S1 Appendix in** S1 File). TPB postulates that behavior is influenced predominantly by individual attitudes, subjective norms, and perceived behavioral control [9]. These constructs were adapted to evaluate them specifically for COVID-19 self-isolation and protective behaviors. An additional four questions were included for attention check purposes (**S1 Appendix in** S1 File). Participants who completed the pre-test survey and correctly answered the four attention-check questions were invited to complete the main Qualtrics survey. To contact prior participants, we used the behavioral research tool, TurkPrime.

The main survey randomized participants to one of three hypothetical scenarios. Each scenario began with the participant experiencing symptoms commonly associated with COVID-19 (fever and cough) and a physician clinically diagnosing them with COVID-19 and advising the participant to self-isolate in accordance with CDC guidelines [10]. Each scenario differed in testing result: in Scenario 1 COVID-19 confirmatory testing was not available, in Scenario 2 the participant received a positive confirmatory test for the COVID-19 virus, and in Scenario 3 the participant received a negative confirmatory test for the COVID-19 virus.

After being presented with their respective scenarios, participants were then asked about their likelihood to engage in a number of behaviors over the following two weeks. These behaviors were primarily selected from those that the CDC identified as increasing the risk for contracting COVID-19 (hereinafter referred to as “risky behaviors”), but also included other risky behaviors of interest to our research team (**S2 Appendix in** S1 File) [11–16]. An additional two attention check questions were included in the main survey (**S2 Appendix in** S1 File). Participants were compensated $0.10 for completing the pre-survey and $0.60 following survey completion.

Two pilot studies were run prior to launching the main survey to estimate power and effect size, as well as validate the internal consistency of the subscales (**S1 Table in** S1 File). The first pilot study results were used to develop an aggregate, 11-item risky behavior score, along with personal decisions and social expectations subscores. Three additional items (voting, protesting/counter-protesting, public transportation) were evaluated as individual scores. The second pilot was used to estimate an effect size (Cohen’s d) for comparing the testing not available condition with the testing positive condition in terms of the 11-item total score. The sample size of 1,194 (398 per condition) was chosen so as to provide 80% power to detect the estimated effect size of 0.23, assuming a two-sample t-test and a two-sided significance level of 0.017 (3-fold Bonferroni correction for pairwise comparison of study scenarios). Survey responses for the main study were collected between July 23^rd^, 2020 to July 29^th^, 2020.

Participants were excluded from the main study analysis if they were unable to complete the English language consent form, did not list a valid U.S. zip code, failed any one of the attention check questions in either the pre-test or the main survey, or completed the main survey in under 120 seconds—a threshold determined by the study team after pre-testing 15 college-educated individuals (**S3 Appendix in** S1 File).

### Statistical analysis

Survey responses were summarized for the full sample and stratified by testing scenario. Quantitative responses were summarized using means, standard deviations and quartiles, and categorical and ordinal responses were summarized using frequency distributions. Covariates included in the regression analysis were pre-specified before the study was carried out.

The analysis consisted of two components: a pre-specified analysis and an exploratory analysis, both consisting of a single set of regressions. All results presented (with the exception of the Theory of Planned Behavior-specific model) are derived from these two groups of regressions.

The primary outcome was the 11-item mean score, ranging from 1 (minimal intention to engage in high-risk behavior) to 7 (maximal intention). Secondary outcomes were the personal decisions and social expectations subscales (each also ranging from 1 to 7), as well as the likelihood of voting, protesting/political gathering, and utilizing public transportation 1-item questions. Each outcome was analyzed using two groups of regressions, with the composite score modeled as a dependent variable. A pre-specified multivariable model included the covariates age, sex, race/ethnicity, political affiliation, education level, location, and type of residence based on results from prior literature [5, 7, 12–16]. A post-hoc model based on univariate associations included as additional covariates self-rated health status, the Consumer Finance Protection Bureau’s (CFPB) financial well-being score (financial well-being score), composite TPB score, region (metro adjacent v. not), and household risk. The financial well-being score is a 5-item score utilized by the U.S. government’s Consumer Finance Bureau to evaluate financial well-being [17]. Given the relative dearth of published behavioral research on COVID-19 spreading behaviors at the time of survey development, the six construct questions were adapted from previous, similar TPB studies of protective behaviors during infectious outbreaks [9, 18, 19]. We ran a confirmatory factor analysis to test these six items for internal validity and found an acceptable Cronbach’s alpha, α = .83; as such, they were aggregated into a single composite we refer to as the composite TPB score. For the pre-registered analysis of the primary outcome, pairwise comparisons of the 3 scenarios were performed using an 0.017 significance level (3-fold Bonferroni correction for an overall alpha of 0.05) [20]. All other analyses applied an 0.05 significance level. All analyses were performed using R v. 3.6.2 (http://www.r-project.org).

## Results

### Participants

Out of 1400 participants who completed the questionnaire, 1194 (85.3%) met all inclusion criteria and were included in the analysis. Scenario 1 (testing unavailable group) contained 401 participants, Scenario 2 (positive confirmatory test group) contained 390 participants, and Scenario 3 (negative confirmatory test group) contained 403 participants (Table 1).

**Table 1:** Participant demographics.

Participant Demographics					
		All Respondents (N = 1194)	Scenario 1—Testing Unavailable (N = 401)	Scenario 2—Positive Test (N = 390)	Scenario 3 –Negative Test (N = 403)
Gender					
	Male	497 (41.6%)	159 (39.7%)	169 (43.3%)	169 (41.9%)
	Female	682 (57.1%)	237 (59.1%)	216 (55.4%)	229 (56.8%)
	Prefer to self-describe	10 (0.8%)	3 (0.7%)	4 (1.0%)	3 (0.7%)
	Prefer not to say	5 (0.4%)	2 (0.5%)	1 (0.3%)	2 (0.5%)
Hispanic Ethnicity					
	Yes	110 (9.2%)	38 (9.5%)	39 (10.0%)	33 (8.2%)
	No	1069 (89.5%)	358 (89.3%)	346 (88.7%)	365 (90.6%)
	Prefer not to say	15 (1.3%)	5 (1.2%)	5 (1.3%)	5 (1.2%)
Race					
	White	911 (79.0%)	295 (76.6%)	303 (80.2%)	313 (80.3%)
	Black or African American	91 (7.9%)	37 (9.6%)	27 (7.1%)	27 (6.9%)
	Asian	105 (9.1%)	39 (10.1%)	33 (8.7%)	33 (8.5%)
	Native Hawaiian or Pacific Islander	2 (0.2%)	1 (0.3%)	1 (0.3%)	0
	American Indian or Alaskan Native	10 (0.9%)	2 (0.5%)	2 (0.5%)	6 (1.5%)
	Some other race, ethnicity, or origin	20 (1.7%)	6 (1.6%)	8 (2.1%)	6 (1.5%)
	Prefer not to say	14 (1.2%)	5 (1.3%)	4 (1.1%)	5 (1.3%)
	Missing	41	16	12	13
Political Affiliation					
	Republican	277 (23.2%)	86 (21.4%)	78 (20.0%)	113 (28.0%)
	Democrat	528 (44.2%)	177 (44.1%)	175 (44.9%)	176 (43.7%)
	Independent	389 (32.6%)	138 (34.4%)	137 (35.1%)	114 (28.3%)
Level of Education					
	8th grade or less	1 (0.1%)	1 (0.2%)	0	0
	Some high school, but did not graduate	8 (0.7%)	4 (1.0%)	3 (0.8%)	1 (0.2%)
	High school graduate or GED	131 (11.0%)	49 (12.2%)	42 (10.8%)	40 (9.9%)
	Some college or 2-year degree	355 (29.7%)	109 (27.2%)	124 (31.8%)	122 (30.3%)
	4-year college degree	451 (37.8%)	155 (38.7%)	130 (33.3%)	166 (41.2%)
	More than 4-year college degree	248 (20.8%)	83 (20.7%)	91 (23.3%)	74 (18.4%)
Type of Residence					
	House/condo/townhouse	910 (76.2%)	313 (78.1%)	298 (76.4%)	299 (74.2%)
	Apartment	271 (22.7%)	83 (20.7%)	88 (22.6%)	100 (24.8%)
	Dormitory	1 (0.1%)	0	0	1 (0.2%)
	Assisted living facility	1 (0.1%)	1 (0.2%)	0	0
	Other	11 (0.9%)	4 (1.0%)	4 (1.0%)	3 (0.7%)
Shared Living Space					
	I live by myself	186 (15.6%)	66 (16.5%)	62 (15.9%)	58 (14.4%)
	2 people	406 (34.0%)	127 (31.7%)	150 (38.5%)	129 (32.0%)
	3 people	243 (20.4%)	84 (20.9%)	75 (19.2%)	84 (20.8%)
	4 people	212 (17.8%)	74 (18.5%)	57 (14.6%)	81 (20.1%)
	5 people	97 (8.1%)	28 (7.0%)	32 (8.2%)	37 (9.2%)
	6 or more people	50 (4.2%)	22 (5.5%)	14 (3.6%)	14 (3.5%)
Overall Health					
	Excellent	183 (15.3%)	73 (18.2%)	48 (12.3%)	62 (15.4%)
	Very good	491 (41.1%)	166 (41.4%)	173 (44.4%)	152 (37.7%)
	Good	382 (32.0%)	111 (27.7%)	126 (32.3%)	145 (36.0%)
	Fair	119 (10.0%)	46 (11.5%)	36 (9.2%)	37 (9.2%)
	Poor	19 (1.6%)	5 (1.2%)	7 (1.8%)	7 (1.7%)
Household Member under 18					
	Yes	419 (35.1%)	147 (36.7%)	117 (30.0%)	155 (38.5%)
	No	775 (64.9%)	254 (63.3%)	273 (70.0%)	248 (61.5%)
Household Risk					
	Yes	353 (29.6%)	128 (31.9%)	112 (28.7%)	113 (28.0%)
	No	829 (69.4%)	268 (66.8%)	274 (70.3%)	287 (71.2%)
	Do not know	7 (0.6%)	3 (0.7%)	3 (0.8%)	1 (0.2%)
	Prefer not to say	5 (0.4%)	2 (0.5%)	1 (0.3%)	2 (0.5%)

Study participant breakdown according to demographic factors and a number of other variables. Proportion of participants relative to the total number of participants is listed in parentheses. Questions corresponding to variables can be found in **S2 Appendix in** S1 File.

### Relationship of COVID-19 test result to behavioral intentions: Primary outcome

**Fig 1:**
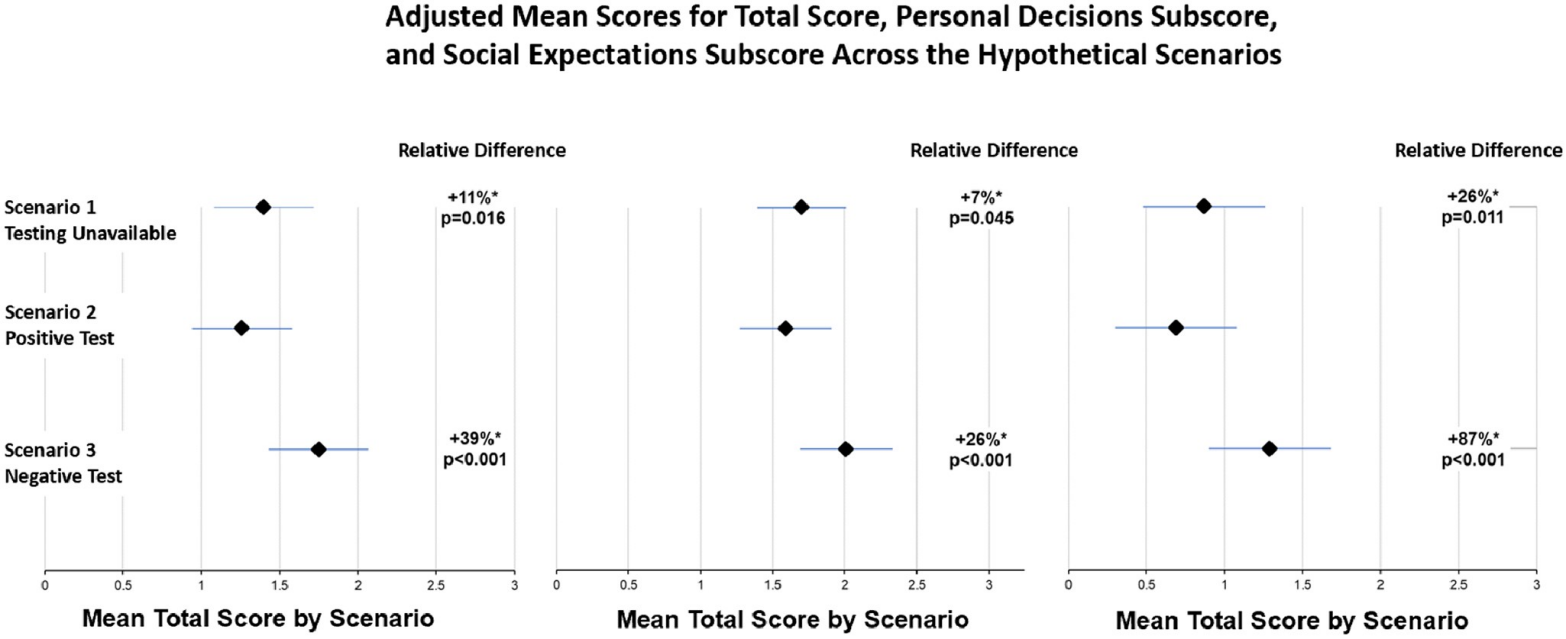
Adjusted mean scores for total score, personal decisions subscore, and social expectations subscore across the hypothetical scenarios.

The analysis of the primary outcome (Tables 2 and 3) indicated that testing unavailability (Scenario 1) resulted in a significantly greater self-reported intention to engage in risky behavior compared to those with a positive test (scenario 2) (mean absolute difference (SE): 0.14 (0.06), *P* = 0.016). This difference corresponds to an 11% relative increase in risky behavior intentions based on mean intention scores (Fig 1). Participants with negative tests demonstrated the greatest intention to engage in risky behavior compared to those without available testing (mean absolute difference (SE): 0.35 (0.06), *P*<0.001) and positive tests (mean absolute difference (SE): 0.49 (0.06), *P*<0.001), respectively. Relative to positive test group’s mean score, those who received a negative test were 39% more likely to engage in risky behavior. Similar significant differences were noted when comparing the personal decisions and the social expectations subscores (Fig 1).

**Table 2:** Linear regression model evaluating associations between behavioral intentions and pre-specified covariates.

	Total Score (R^2^ = 0.11)			Personal Score (R^2^ = 0.10)			Social Score (R^2^ = 0.10)		
Effect	Mean Estimate	SE	P	Mean Estimate	SE	P	Mean Estimate	SE	P
Scenario 2 v. 1	-0.14	0.06	**0.016**	-0.12	0.06	**0.045**	-0.18	0.07	**0.011**
Scenario 3 v. 1	0.35	0.06	**<0.001**	0.31	0.06	**<0.001**	0.42	0.07	**<0.001**
Scenario 3 v. 2	0.49	0.06	**<0.001**	0.42	0.06	**<0.001**	0.60	0.07	**<0.001**
Age (+1y)	0.00	0.00	**0.036**	0.00	0.00	0.177	-0.01	0.00	**0.006**
Gender (Ref = Male)			**0.024**			**0.011**			0.137
Female	-0.13	0.05	**0.009**	-0.15	0.05	**0.002**	-0.09	0.06	0.147
Prefer to self-describe	-0.34	0.29	0.228	-0.25	0.28	0.371	-0.50	0.35	0.157
Prefer not to say	-0.65	0.42	0.124	-0.59	0.42	0.162	-0.75	0.52	0.148
Race/Ethnicity (Ref = White)			0.854			0.316			0.897
Hispanic	-0.06	0.09	0.458	-0.09	0.09	0.289	-0.02	0.11	0.875
Black or African American	0.04	0.09	0.633	0.10	0.09	0.266	-0.06	0.12	0.613
Asian	-0.04	0.09	0.607	-0.12	0.09	0.150	0.09	0.11	0.376
Native Hawaiian or Pacific Islander	0.42	0.80	0.596	0.94	0.79	0.239	-0.47	0.99	0.632
American Indian or Alaskan Native	-0.37	0.33	0.261	-0.34	0.33	0.291	-0.41	0.41	0.313
Some other race, ethnicity, or origin	-0.06	0.30	0.837	0.00	0.30	0.995	-0.17	0.38	0.654
Prefer not to say	0.21	0.27	0.447	0.23	0.27	0.385	0.16	0.34	0.641
Political Affiliation (Ref = Republican)			**<0.001**			**<0.001**			**<0.001**
Democrat	-0.36	0.06	**<0.001**	-0.32	0.06	**<0.001**	-0.42	0.08	**<0.001**
Independent	-0.13	0.07	**0.041**	-0.15	0.07	**0.021**	-0.10	0.08	0.197
Education (Ref = 8th grade or less)			0.587			0.445			0.861
Some high school, but did not graduate	0.07	0.85	0.935	-0.20	0.84	0.816	0.53	1.05	0.611
High school graduate or GED	0.07	0.80	0.929	-0.17	0.80	0.835	0.49	0.99	0.623
Some college or 2-year degree	0.16	0.80	0.838	-0.07	0.79	0.930	0.57	0.99	0.562
4-year college degree	0.14	0.80	0.865	-0.11	0.79	0.891	0.56	0.99	0.568
More than 4-year college degree	0.23	0.80	0.775	0.00	0.80	0.999	0.62	0.99	0.528
Residence (Ref = House/condo/townhouse)			0.807			0.600			0.931
Apartment	-0.07	0.06	0.228	-0.09	0.06	0.111	-0.03	0.07	0.661
Dormitory	-0.24	0.80	0.770	0.10	0.80	0.901	-0.82	1.00	0.410
Assisted Living Facility	-0.10	0.80	0.898	-0.16	0.80	0.843	-0.01	0.99	0.995
Other	-0.08	0.26	0.747	-0.12	0.26	0.641	-0.02	0.32	0.952

Pre-specified multi-variable regression analysis based on the pre-specified covariates. Pre-specified variables included: age, sex, race/ethnicity, political affiliation, education level, location, and type of residence. Mean estimate refers to mean absolute estimate difference compared to the reference group, and SE refers to Standard Error. Statistically significant differences were shown through bolded p-values. R^2^ refers to the amount of variance within the dependent variable that is explained by the independent variables in the regression model.

**Table 3:** Post-hoc linear regression model evaluating associations between behavioral intentions (total score, subscores, voting, and protest) and additional covariates.

	Total Score (R^2^ = 0.35)			Personal Score (R^2^ = 0.32)			Social Score (R^2^ = 0.29)			Voting (R^2^ = 0.16)			Protest (R^2^ = 0.15)			Transportation (R^2^ = 0.04)		
Effect	Mean Estimate	SE	P	Mean Estimate	SE	P	Mean Estimate	SE	P	Mean Estimate	SE	P	Mean Estimate	SE	P	Mean Estimate	SE	P
Scenario 2 v. 1	-0.17	0.05	**<0.001**	-0.15	0.05	**0.003**	-0.22	0.06	**<0.001**	-0.33	0.12	**0.006**	-0.11	0.04	**0.012**	0.10	0.11	0.374
Scenario 3 v. 1	0.32	0.05	**<0.001**	0.28	0.05	**<0.001**	0.38	0.06	**<0.001**	0.39	0.12	**0.001**	0.11	0.04	**0.011**	-0.06	0.11	0.592
Scenario 3 v. 2	0.50	0.05	**<0.001**	0.44	0.05	**<0.001**	0.60	0.06	**<0.001**	0.73	0.12	**<0.001**	0.22	0.04	**<0.001**	-0.16	0.11	0.156
Age (+1y)	0.00	0.00	0.792	0.00	0.00	0.596	0.00	0.00	0.199	0.01	0.00	0.091	0.00	0.00	0.408	0.00	0.00	0.277
Gender (Ref = Male)			0.130			0.105			0.213			0.534			0.788			0.248
Female	-0.06	0.04	0.163	-0.08	0.04	**0.047**	-0.01	0.05	0.798	-0.13	0.10	0.191	0.03	0.04	0.348	0.10	0.09	0.275
Prefer to self-describe	-0.38	0.25	0.121	-0.29	0.25	0.243	-0.55	0.32	0.088	-0.27	0.60	0.651	0.09	0.21	0.688	-0.46	0.56	0.411
Prefer not to say	-0.54	0.38	0.157	-0.49	0.38	0.203	-0.63	0.49	0.202	-0.67	0.93	0.473	-0.07	0.33	0.836	-1.16	0.86	0.178
Race/Ethnicity (Ref = White)			0.725			0.176			0.798			0.957			0.798			0.787
Hispanic	-0.07	0.07	0.346	-0.10	0.08	0.208	-0.03	0.10	0.773	-0.20	0.18	0.281	0.04	0.07	0.546	0.11	0.17	0.511
Black or African American	0.05	0.08	0.554	0.11	0.08	0.192	-0.05	0.10	0.602	-0.05	0.19	0.783	0.01	0.07	0.919	0.00	0.18	0.986
Asian	0.00	0.07	0.963	-0.07	0.07	0.322	0.14	0.10	0.147	-0.15	0.18	0.397	0.02	0.06	0.716	0.15	0.17	0.384
Native Hawaiian or Pacific Islander	0.84	0.69	0.220	1.35	0.69	0.052	-0.04	0.89	0.961	-0.43	1.67	0.797	0.10	0.60	0.864	-0.83	1.55	0.593
American Indian or Alaskan Native	-0.34	0.28	0.226	-0.34	0.29	0.238	-0.35	0.37	0.335	-0.28	0.69	0.688	0.06	0.25	0.804	-0.39	0.64	0.541
Some other race, ethnicity, or origin	0.06	0.26	0.817	0.13	0.26	0.618	-0.06	0.34	0.852	0.06	0.64	0.926	0.13	0.23	0.557	0.52	0.59	0.379
Prefer not to say	0.03	0.24	0.894	0.05	0.24	0.841	0.00	0.30	0.991	0.17	0.57	0.760	0.37	0.20	0.074	0.68	0.53	0.205
Political Affiliation (Ref = Republican)			0.082			0.364			**0.014**			0.437			0.135			0.551
Democrat	-0.10	0.06	0.089	-0.07	0.06	0.199	-0.14	0.07	0.061	-0.10	0.14	0.452	0.00	0.05	0.958	-0.13	0.13	0.287
Independent	0.00	0.06	0.960	-0.02	0.06	0.737	0.04	0.07	0.572	-0.18	0.14	0.199	0.08	0.05	0.113	-0.11	0.13	0.389
Education (Ref = 8th grade or less)			0.234			0.233			0.423			0.208			0.848			0.573
Some high school, but did not graduate	0.69	0.73	0.348	0.39	0.74	0.602	1.22	0.95	0.199	1.21	1.78	0.497	0.49	0.64	0.445	0.95	1.66	0.568
High school graduate or GED	0.53	0.69	0.445	0.26	0.70	0.705	0.99	0.90	0.268	1.03	1.68	0.542	0.45	0.60	0.458	0.47	1.57	0.762
Some college or 2-year degree	0.66	0.69	0.339	0.39	0.70	0.579	1.14	0.90	0.202	1.27	1.68	0.449	0.40	0.60	0.504	0.64	1.57	0.684
4-year college degree	0.61	0.69	0.376	0.33	0.70	0.639	1.11	0.90	0.214	1.33	1.68	0.428	0.40	0.60	0.503	0.76	1.57	0.628
More than 4-year college degree	0.70	0.69	0.312	0.44	0.70	0.533	1.17	0.90	0.193	1.50	1.68	0.372	0.45	0.60	0.457	0.65	1.57	0.679
Health Status (Ref = "Excellent")			**0.037**			**0.028**			**0.033**			**0.026**			0.947			0.801
Very good	-0.13	0.06	**0.037**	-0.06	0.06	0.330	-0.25	0.08	**0.002**	-0.46	0.15	**0.002**	0.03	0.05	0.613	-0.04	0.14	0.759
Good	-0.15	0.07	**0.021**	-0.11	0.07	0.104	-0.23	0.08	**0.007**	-0.37	0.16	**0.018**	0.03	0.06	0.649	0.09	0.15	0.557
Fair	-0.09	0.09	0.307	-0.03	0.09	0.739	-0.20	0.11	0.086	-0.44	0.21	**0.040**	-0.01	0.08	0.897	-0.04	0.20	0.845
Poor	-0.45	0.17	**0.008**	-0.52	0.17	**0.003**	-0.33	0.22	0.135	-0.81	0.42	0.051	-0.04	0.15	0.782	0.15	0.39	0.697
CFPB Score (+1)	0.00	0.00	0.635	0.00	0.00	0.431	0.00	0.00	0.947	0.00	0.00	0.555	0.00	0.00	0.342	0.00	0.00	0.975
Construct Score (+1)	-0.12	0.01	**<0.001**	-0.11	0.01	**<0.001**	-0.12	0.01	**<0.001**	-0.16	0.01	**<0.001**	-0.06	0.01	**<0.001**	-0.07	0.01	**<0.001**
Description (Ref = Metro)			**0.025**			0.082			**0.022**			0.562			0.220			0.747
Nonmetro—metro adjacent	0.21	0.08	**0.007**	0.17	0.08	**0.029**	0.28	0.10	**0.006**	0.20	0.19	0.309	0.11	0.07	0.109	0.00	0.18	0.986
Nonmetro—not metro adjacent	0.06	0.10	0.534	0.07	0.10	0.516	0.06	0.13	0.661	0.11	0.25	0.670	0.07	0.09	0.419	-0.17	0.23	0.448
Household Risk (Ref = Yes)			0.110			0.113			0.190			0.476			0.884			0.336
No	0.09	0.05	0.054	0.07	0.05	0.132	0.13	0.06	**0.040**	-0.14	0.12	0.226	0.00	0.04	0.927	0.18	0.11	0.089
Do not know	0.36	0.27	0.179	0.48	0.27	0.078	0.16	0.35	0.646	0.56	0.65	0.393	-0.09	0.23	0.686	-0.22	0.61	0.721
Prefer not to say	0.43	0.34	0.210	0.43	0.34	0.206	0.42	0.44	0.345	-0.16	0.83	0.842	-0.21	0.30	0.479	0.46	0.77	0.553
Residence (Ref = House/condo/townhouse)			0.200			0.097			0.684			0.182			0.589			0.609
Apartment/assisted living facility/dormitory	-0.09	0.05	0.078	-0.11	0.05	**0.033**	-0.06	0.07	0.393	-0.20	0.12	0.108	-0.04	0.04	0.411	0.11	0.11	0.322
Other	-0.09	0.22	0.674	-0.11	0.23	0.619	-0.06	0.29	0.827	-0.54	0.54	0.317	-0.13	0.19	0.506	0.08	0.51	0.878

Post-hoc linear regression analysis, which includes additional covariates (health status, zip code density, CFPB, and composite TPB score) compared to the pre-specified multivariable model. Mean estimate refers to mean absolute estimate difference compared to the reference group, and SE refers to Standard Error. Statistically significant differences were shown through bolded p-values. CFPB refers to Consumer Finance Protection Bureau’s (CFPB) financial well-being score. Construct score refers to the composite TPB score. R^2^ refers to the amount of variance within the dependent variable that is explained by the independent variables in the regression model.

The diamond represents mean behavioral intention scores based on primary, personal decision, and social expectations subscores. Bars represent Standard Errors. Y-axis depicts specific scenarios and x-axis depicts mean behavioral intentions based on 7-point Likert scale. “Relative Difference” refers to the relative difference in mean behavioral intention scores with Arm 2’s mean score as a reference. Asterisks after the “Relative Difference” number indicate that the mean difference was found to be statistically significant compared to Arm 2 (indicated by adjacent p-values).

### Linear regression modelling

The pre-specified multivariable model (Table 2) included several covariates (gender, race, ethnicity, political affiliation, level of education, and type of residence) and explained only 11% of the variance in the primary outcome. As a result, a post-hoc model (Table 3) incorporating several additional covariates (health status, financial well-being, composite TPB score, metro code, and household risk) was developed in order to gain a better understanding of the factors driving risky behavior and was found to explain 35% of the primary outcome variance. Exploratory analyses suggest that the R^2^ of the pre-specified model was largely driven by scenario differences and political affiliation, while the R^2^ for the post-hoc model was largely driven by scenario differences, political affiliation, and composite TPB scores.

### Gender

When compared to those who identify as men, those identifying as women reported significantly decreased risky behavior intentions (mean absolute difference (SE): -0.13 (0.05), *P* = 0.009) (Table 2). However, in the post-hoc model that included a control for pre-existing attitudes about the disease (the composite TPB score), this gender difference in behavior intentions was non-significant (mean absolute difference (SE): -0.06 (0.04), *P* = 0.163) (Table 3).

### Health status

After controlling for other variables in the post-hoc model, self-identified health status was found to be significantly associated with behavioral intentions. Compared to those who reported “excellent” health status, those who reported a “poor” health status expressed the lowest intentions to engage in risky behavior (difference (SE): -0.45 (0.17), *P* = 0.008) (Table 3).

### Political affiliation

In the pre-specified model, Republicans reported higher intentions to engage in risky behavior than either Democrats (mean absolute difference (SE): 0.36 (0.06), *P*<0.001) or Independents (mean absolute difference (SE): -0.13 (0.07), *P* = 0.041), respectively (Table 2). Specifically, Republicans showed a 27% relative increase in mean intention score to engage in risky behavior compared to Democrats. Republicans were also less likely than Democrats and Independents to agree with the statement that “COVID-19 could have severe consequences on other peoples’ lives”. In the post-hoc model that included a control for pre-existing attitudes about the severity of the disease, the effect of political identity on intention to self-isolate was no longer significant (Table 3).

### Voting and rally / Protest intentions

Given the survey’s timing during a politically tumultuous year and several months before the 2020 United States presidential election, we assessed respondents’ voting intentions and intention to participate in a large-scale political event (a rally or a protest) using our pre-specified regression model (**S3 Table in** S1 File). Participants who received a positive test result indicated a lower intention to vote in person than participants who had not received confirmatory testing (mean absolute difference (SE): -0.30 (0.13), *P* = 0.018). Participants who tested negative indicated higher intentions to participate in a rally or protest than both participants who tested positive (mean absolute difference (SE): 0.21 (0.05), *P*<0.001) and participants who did not receive a test result (mean absolute difference (SE): 0.13 (0.05), *P* = 0.005).

Political affiliation also affected intention to vote in-person and intention to attend a rally or protest. Democrats reported decreased intentions to cast a ballot at a voting station (mean absolute difference (SE): -0.43 (0.14), *P* = 0.002) and attend a rally, protest or counter-protest (mean absolute difference (SE): -0.13 (0.05), *P* = 0.008) compared to Republican respondents. Translating the mean absolute differences to relative decreases, Democrats exhibited 33% and 12% lower intention scores to vote in-person and attend a protest/political rally, respectively. Those identifying as politically independent indicated significantly decreased intentions to vote in-person (mean absolute difference (SE): -0.34 (0.14), *P* = 0.017) compared to their Republican counterparts, but were not significantly less likely to attend a rally or a protest.

### The theory of planned behavior

Given the notable R^2^ difference between the pre-specified model (Table 2) and that of the exploratory model (Table 3), an additional post-hoc, TPB-specific linear regression model composed of the six individual TPB questions was constructed (**S4 Table in** S1 File). This TPB-specific R^2^ value (R^2^ = 0.36) suggests that the composite TPB score plays a significant role in explaining the variance seen with the total score. When evaluated within this TPB-specific, post-hoc model, the first subjective norms question, “*People with COVID-19 should self-isolate*” showed the largest negative association with risky behavioral intentions (mean absolute difference (SE): -0.27 (0.05), *P*<0.001) (**S4 Table in** S1 File). Both questions assessing preconceived attitudes about COVID-19 demonstrated significant, yet smaller effect sizes (mean absolute difference (SE): -0.12 (0.05), *P* = 0.009) (mean absolute difference (SE): -0.15 (0.03), *P*<0.001), and perceived behavioral control demonstrated a weaker, but nonetheless significant effect (mean absolute difference (SE): -0.05 (0.02), *P* = 0.022).

## Discussion

In this randomized hypothetical scenario study, participants who were clinically diagnosed with COVID-19 but had no testing available to them exhibited an 11% relative increase in intention to engage in risky behavior compared to those with a positive confirmatory test. Additionally, clinically symptomatic participants who received a negative test reported higher intentions to engage in risky behavior than any other group.

Although significant advancements (e.g., increased testing capacity, several effective COVID-19 vaccines) have been made since start of the COVID-19 pandemic, our study’s findings continue to be relevant not only due to the continued outbreaks and transmission of the virus, but also because decreases in COVID-19 testing rates made it more difficult to identify surges in real time [21, 22]. While COVID-19 vaccines have conferred substantial protection from severe symptoms, uneven vaccine uptake coupled with the recent drastic increase in testing positivity rates nationwide (11.5% as of August 2021) suggest that increased testing still has a major role in combating viral transmission [23, 24].

To the best of our knowledge, this study is the first to demonstrate how testing unavailability independently decreases intention to isolate in patients clinically diagnosed with COVID-19. While increasing testing availability alone will not fully eliminate viral transmission, previous literature indicates that relatively small degrees of behavioral change (e.g., decreasing visits to non-essential businesses, wearing masks) may result in major decreases in viral transmission [25, 26]. Thus, while the magnitude of behavior intention change reported in this study is small on an absolute level, our findings nonetheless suggest a clear role that testing availability could play in curbing viral transmission.

In addition to the impact of testing unavailability, it is interesting to note the impact a negative test has on increasing risky behavior intentions. Despite a clinical diagnosis, those with a negative confirmatory test were significantly more likely to engage in behaviors facilitating viral transmission, likely because they believed that they could not transmit the virus to others. While a negative COVID-19 test does certainly reduce one’s likelihood of having an active COVID-19 infection, diagnostic tests—particularly rapid antigen tests—are widely acknowledged to yield false-negative results in approximately 10–15% of cases and are dependent on when they are administered during the illness course [27–29]. Furthermore, one recent analysis of COVID-19 testing policies and subsequent COVID-19 disease burden suggested that areas which implemented testing-on-demand policies subsequently had the greatest COVID-19 hospitalization rates, highlighting the potential behavioral externalities associated with negative test results and complicating the question of whether increased testing uniformly results in decreased public health risk [30]. These results have been similarly reported within HIV literature, where several studies have suggested that receiving an HIV-negative test can be associated with a possible increase in future sexual risk behavior; however, these behavioral effects appear to be heterogeneous based on personal expectation [31].

The present results are also notable for the effect that political leanings have on the intention to self-isolate. Prior studies have found a correlation between mistrust of government-issued guidelines and partisan affiliation [32]. This study finds that Republicans are not only less likely to agree that “*COVID-19 could have severe consequences on other peoples’ lives*,”, but also are 27% more likely to engage in risky behaviors compared to Democrats based on mean intention scores. These findings indicate that decreased belief in the dangers of the disease may play a role in politically-related decisions to self-isolate, and may suggest a potential pathway by which partisan beliefs influence behavioral intentions.

The results presented here also suggest that political leanings affect both intentions to vote in-person and intentions to attend political events that involve crowds (e.g., rally, protest). Regardless of testing availability, Democrats with a presumed COVID-19 diagnosis indicated a remarkable 33% lower likelihood to engage in in-person voting based on their respective mean intention scores. At the time of our initial analysis, one downstream implication of these exploratory results was that communities burdened with high rates of COVID-19 infections during Election Day might have significantly lower Democratic than Republican on-site turnout. Indeed, the intention differences observed in our study trend similarly to the in-person voting turnout rates seen during the 2020 national election, where only 41% of Democrats reported voting in-person, compared to only 70% of Republicans [33]. Presuming that the current pandemic continues or a subsequent infectious outbreak from another contagion occurs during a future election cycle, it is critical to recognize and accommodate for these stark voting preferences in order to ensure that electoral processes occur fairly and equitably.

We found that those with poorer perceived health status revealed greater intentions to self-isolate, regardless of study scenario. These findings are consistent with previous hypothetical scenario studies suggesting that increased risk perception is associated with adopting self-protective behaviors. To our knowledge, this is the first hypothetical scenario study to evaluate the behavioral intention effects in a post-infection scenario [34–36].

### Limitations

There are several limitations to our study. First, survey respondents were recruited using MTurk, an online platform that—while as effective as traditional survey sampling methods—skews towards younger, more well-educated individuals [37]. Although this affects the generalizability of our sample, the risks of in-person surveys at the time this study was conducted outweighed the benefits.

Study findings may also be limited by the hypothetical nature of the survey design. Although few studies have evaluated the extent of hypothetical bias specifically in stated choice experiments, it is very possible that there is some discrepancy between participants’ stated choices and what they would decide in real-life decisions [38]. Similarly, it is possible that participants may have underestimated how likely they would engage in negatively perceived behaviors, resulting in some desirability bias. However, survey scenarios were emphasized to be anonymous, designed to be easily readable, and were repeated on several pages in order to facilitate participant comprehension and immersion (**S4 Appendix in** S1 File). In addition, survey response options for the behavior questions incorporated certainty scales (e.g. extremely unlikely to extremely likely), a mitigation strategy thought to reduce hypothetical bias. Furthermore, to reduce the number of participants who did not take an appropriate amount of time to imagine the scenario, we restricted analysis to participants who spent at least 2 minutes on the survey and passed all attention checks.

External validity of this study is limited by the fact that the study only evaluated behavioral intentions. A large body of prior research has noted that behavioral intentions do not immediately translate to behavioral engagement but are rather attenuated or enhanced by other factors. Nevertheless, behavioral intentions are commonly acknowledged as one of the best predictors of behaviors themselves, so it is likely that differences in behavior between the different groups would still exist [39, 40].

Although not directly evaluated here, it may be interesting to consider the effect of waiting to receive a definitive result on intention to engage in risky behaviors. While we hypothesize that a delay in test results might lead patients to behave similarly to respondents assigned to Scenario 1, further studies are necessary to evaluate the effect of testing delay on self-isolation behavior.

Lastly, given the dynamic nature of the global pandemic and the recent shifts in public opinion towards both the global pandemic and self-protective measures, public opinion on this subject will likely continue to shift over time [41]. Despite these limitations, these significant differences in behavioral intentions are novel findings providing evidence that increased testing capacity may ultimately translate into fewer infections and fewer deaths.

## Conclusion

Testing availability independently influences patients’ intentions to engage in COVID-19 risky behaviors, even when controlling for a clinical diagnosis of COVID-19. Such findings shed light on the potential behavioral externalities associated with both testing unavailability and negative test results, and ultimately highlight the role of testing may play in influencing the public’s behavioral response to future contagions.

## Supporting information

S1 File (DOCX)
